# Supplementary material for: Dynamics of Transformation from Segregation to Mixed Wealth Cities
Source: PLoS One. 2016 Nov 18;11(11):e0166960. doi: 10.1371/journal.pone.0166960 (PMC5115835; doi:10.1371/journal.pone.0166960)
Supplement: S1 Appendix — (PDF) [file pone.0166960.s001.pdf]

S1 Appendix. Impact of τ on emergence of segregation. In order to to understand the onset of segregation with varying τ, we start with defining a threshold for the emergence of segregation. Given that a Moore neighborhood implies eight neighbors, we define an average Size of Rich Neighborhood of 1.5 as the threshold for segregation. Below this threshold, the spatial distribution is defined to be unsegregated, and this is justifiable by the fact that the average Size of Rich Neighborhoods (S) for a spatially mixed (random) distribution is approximately 1.3. With this definition in place, we study the dynamics for all values of τ between 1 and 7 at $\beta = 100$ (which corresponds to zero disallowed-realized moves). We run 25 iterations for each value of τ.

As Fig 1 clearly demonstrates, we find that segregation occurs with increasing intensity as τ increases and is maximized at $\tau = 4$, but for $\tau \geq 5$ segregation does not emerge at all. This is potentially explained by the fact that increasing the threshold condition τ essentially makes agent swaps progressively more difficult to accomplish, meaning that as τ increases only those agents with wealths high enough to satisfy the (increasing) threshold condition are able to swap out of their lower wealth neighborhoods to higher wealth neighborhoods, yielding higher levels of segregation. However, at very high values ($\tau \geq 5$), the threshold condition becomes so stringent as to prevent almost any moves at all, causing the initial distribution to persist, and since we start with a mixed distribution, segregation does not emerge. Consequently, the dynamics result in a threshold of $\tau = 4$ or a corresponding threshold ratio of 50%.

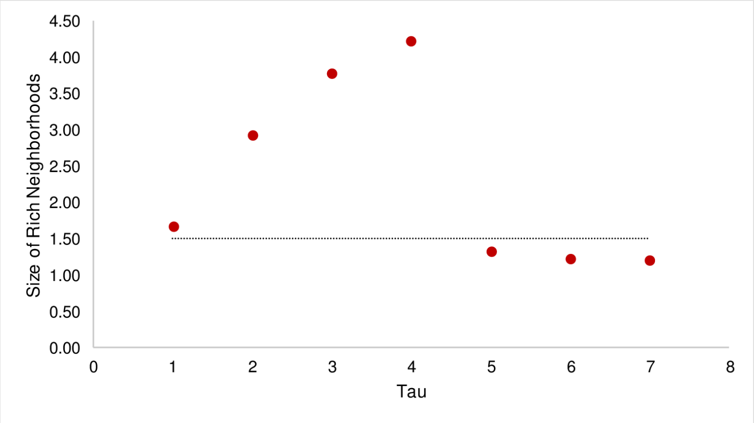

Fig 1. Change in Size of Rich Neighborhoods with τ. Legend: Dashed Line: Threshold for segregation (Size of Rich Neighborhoods = 1.5).
