# Supplementary material for: Dynamics of Transformation from Segregation to Mixed Wealth Cities
Source: PLoS One. 2016 Nov 18;11(11):e0166960. doi: 10.1371/journal.pone.0166960 (PMC5115835; doi:10.1371/journal.pone.0166960)
Supplement: S2 Appendix — (PDF) [file pone.0166960.s002.pdf]

S2 Appendix. Modeling spatial dynamics on an Erdős-Rényi random graph. The graph is created with the following parameters: Number of nodes (agents) = 625, and Link Probability = 4/625. This implies that while nodes have 4 neighbors on average, individual nodes have varying numbers of edges. We run 100,000 iterations for each realization of the dynamics and 25 realizations for each β value. Fig 1 plots segregation measures (Rich Wealth Differential and Fraction of Rich Neighbors - which is the fraction of rich neighbors of a rich agent) as a function of disallowed-realized moves, and despite the lack of geometry and the heterogeneity in neighborhood size, we still see the sharp transformation at low levels of disallowed-realized moves. To be sure, the transformation is not as sharp as observed for the Moore neighborhood, but we observe that at about 11.75% of Disallowed-Realized Moves, the change in Rich Wealth Differential is over 38% and that in Fraction of Rich Neighbors is over 58% of their final values (corresponding to $\beta = 0.001$). Overall, the shapes of the curves correspond with the corresponding plots for the square lattice model with Moore neighborhood.

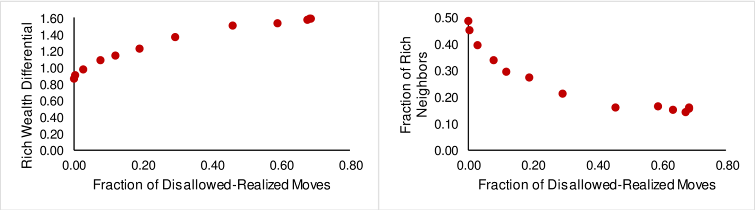

Fig 1. A: Rich Wealth Differential v. Fraction of Disallowed-Realized Moves (left). B: Fraction of Rich Neighbors v. Fraction of Disallowed-Realized Moves (right).
