# Supplementary material for: Dynamics of Transformation from Segregation to Mixed Wealth Cities
Source: PLoS One. 2016 Nov 18;11(11):e0166960. doi: 10.1371/journal.pone.0166960 (PMC5115835; doi:10.1371/journal.pone.0166960)
Supplement: S3 Appendix — (PDF) [file pone.0166960.s003.pdf]

S3 Appendix. Classic Schelling model of wealth based segregation. Spatial dynamics in this model is driven by agent choice modeled as the Tolerance Level (T) in a Moore neighborhood. In each iteration of the classic Schelling model, two agents $\tilde{\mathrm{A}}$ and $\tilde{\mathrm{B}}$ are selected at random and their respective wealths are compared to the wealths of each of the agents in the other's neighborhood. Let $\tilde{\mathrm{n}}_{\mathrm{A}}$ and $\tilde{\mathrm{n}}_{\mathrm{B}}$ be the number of neighbors of $\tilde{\mathrm{A}}(\tilde{\mathrm{B}})$ whose wealths are lesser than that of $\tilde{\mathrm{B}}(\tilde{\mathrm{A}})$ respectively. If both $\tilde{\mathrm{n}}_{\mathrm{A}}$ and $\tilde{\mathrm{n}}_{\mathrm{B}}$ are lesser than or equal to T, then the agents exchange places with probability 1. However, if this is not the case, then an exchange occurs with probability $\tilde{\mathrm{p}}_{\mathrm{m}}$ (Eq 1).

$$\tilde{\mathrm{p}}_{\mathrm{m}} = \exp(\beta\tilde{\Delta}), where : \tilde{\Delta} = -(\tilde{n}_A + \tilde{n}_B) \tag{1}$$

The model uses T = 6, implying that agents are willing to move to a location where no more than 6 of their Moore neighbors have lower wealth than them. We choose T = 6 because this is the value of T at which the highest level of segregation emerges (given $\beta = 100$, which equates to zero disallowed-realized moves), corresponding to $\tau = 4$ in our variant of the model. As β progressively declines from 100 to 0.00001, it essentially implies an increasing occurrence of moves in contravention of the tolerance condition. We run 100,000 iterations for each realization of the dynamics and 20 realizations for each β value. Fig 1 plots segregation measures as a function of disallowed-realized moves, and the shapes of the curves closely match the corresponding plots for our variant of the Schelling model. This is to be expected because given a T that begets segregation, the calibrating factor (β) only progressively increases the ease of moving. More importantly, because the two models are essentially implementations of the same phenomenon with the threshold / tolerance condition being mirror images of each other, we would expect to observe the sharp transformation in both models.

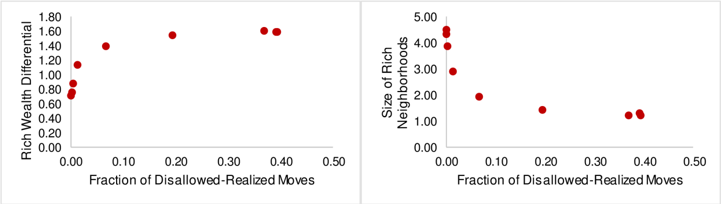

Fig 1. A: Rich Wealth Differential v. Fraction of Disallowed-Realized Moves (left). B: Size of Rich Neighborhoods v. Fraction of Disallowed-Realized Moves (right).

This mirror-imagery is further borne out by Fig 2 which plots the Size of Rich Neighborhoods (S) against T and reveals that segregation in the classic Schelling model emerges and persists for $T \geq 4$, while in our variant of the model, it emerges for $\tau \leq 4$ (S1 Appendix).

It may seem surprising that segregation emerges and increases as the tolerance level increases in the classic Schelling implementation, but an examination of the dynamics reveals that low tolerance levels create extremely stringent conditions for any movement to occur. For instance, given T = 1, an agent swap can occur only if each agent has wealth lesser than at least 7 agents in the other's neighborhood. Therefore, for low T, this stringency ensures that the initial mixed spatial

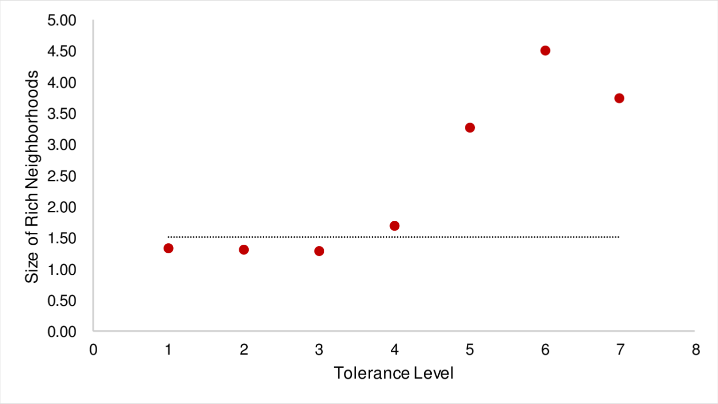

Fig 2. Change in Size of Rich Neighborhoods with Tolerance Level. Legend: Dashed Line: Threshold for segregation (Size of Rich Neighborhoods = 1.5).

distribution persists as there is almost no potential for movement. However, as T increases, and starting at T = 4, we begin to see the possibility of some swaps occurring and the emergence of a low level of segregation. Beyond T = 4, higher levels of segregation obtain and S is maximized at T = 6.
